# A community-based contact isolation strategy to reduce the spread of Ebola virus disease: an analysis of the 2018–2020 outbreak in the Democratic Republic of the Congo

**DOI:** 10.1136/bmjgh-2023-011907

**Published:** 2023-06-01

**Authors:** Mory Keita, Jonathan A Polonsky, Steve Ahuka-Mundeke, Michel Kalongo Ilumbulumbu, Adama Dakissaga, Hamadou Boiro, Julienne Ngoundoung Anoko, Lamine Diassy, John Kombe Ngwama, Houssainatou Bah, Michel Kasereka Tosalisana, Richard Kitenge Omasumbu, Ibrahima Sory Chérif, Samuel T Boland, Alexandre Delamou, Abdoulaye Yam, Antoine Flahault, Stéphanie Dagron, Abdou Salam Gueye, Olivia Keiser, Ibrahima Socé Fall

**Affiliations:** 1 Emergency Preparedness and Response, World Health Organization Regional Office for Africa, Brazzaville, Congo; 2 Institute of Global Health, Faculty of Medicine, University of Geneva, Geneva, Switzerland; 3 Geneva Centre of Humanitarian Studies, University of Geneva, Geneva, Switzerland; 4 Département de Virologie, Institut National de Recherche Biomédicale, Kinshasa, Congo (the Democratic Republic of the); 5 Zone de Santé de Béni, Ministère Provinciale de la Santé, Goma, Congo (the Democratic Republic of the); 6 Direction Régionale de la Santé du Plateau Central, Ministère de la Santé et de l'Hygiène Publique, Ziniaré, Burkina Faso; 7 Direction Générale de la Lutte contre la Maladie, Ministère de la Santé, Kinshasa, Democratic Republic of Congo; 8 Equipe Médicale d'Urgence, Ministère de la Santé Publique, Kinshasa, Congo (the Democratic Republic of the); 9 Country Office for Guinea, World Health Organization, Conakry, Guinea; 10 Centre for Universal Health, Chatham House, London, UK; 11 African Centre of Excellence for the Prevention and Control of Communicable Diseases, Gamal Abdel Nasser University of Conakry, Conakry, Guinea; 12 Global Neglected Tropical Diseases programme, World Health Organization, Geneva, Switzerland

**Keywords:** Viral haemorrhagic fevers, Control strategies, Intervention study, Public Health

## Abstract

**Introduction:**

Despite tremendous progress in the development of diagnostics, vaccines and therapeutics for Ebola virus disease (EVD), challenges remain in the implementation of holistic strategies to rapidly curtail outbreaks. We investigated the effectiveness of a community-based contact isolation strategy to limit the spread of the disease in the Democratic Republic of Congo (DRC).

**Methods:**

We did a quasi-experimental comparison study. Eligible participants were EVD contacts registered from 12 June 2019 to 18 May 2020 in Beni and Mabalako Health Zones. Intervention group participants were isolated to specific community sites for the duration of their follow-up. Comparison group participants underwent contact tracing without isolation. The primary outcome was measured as the reproduction number (R) in the two groups. Secondary outcomes were the delay from symptom onset to isolation and case management, case fatality rate (CFR) and vaccination uptake.

**Results:**

27 324 EVD contacts were included in the study; 585 in the intervention group and 26 739 in the comparison group. The intervention group generated 32 confirmed cases (5.5%) in the first generation, while the comparison group generated 87 (0.3%). However, the 32 confirmed cases arising from the intervention contacts did not generate any additional transmission (R=0.00), whereas the 87 confirmed cases arising from the comparison group generated 99 secondary cases (R=1.14). The average delay between symptom onset and case isolation was shorter (1.3 vs 4.8 days; p<0.0001), CFR lower (12.5% vs 48.4%; p=0.0001) and postexposure vaccination uptake higher (86.0% vs 56.8%; p<0.0001) in the intervention group compared with the comparison group. A significant difference was also found between intervention and comparison groups in survival rate at the discharge of hospitalised confirmed patients (87.9% vs 47.7%, respectively; p=0.0004).

**Conclusion:**

The community-based contact isolation strategy used in DRC shows promise as a potentially effective approach for the rapid cessation of EVD transmission, highlighting the importance of rapidly implemented, community-oriented and trust-building control strategies.

**WHAT IS ALREADY KNOWN ON THIS TOPIC**

- Isolation of cases, quarantines of their close contacts and other forms of physical isolation are approaches to reducing social and physical contact of individuals with the potential to transmit infection.

**WHAT THIS STUDY ADDS**

- A community-based contact isolation strategy implemented in the Democratic Republic of Congo to curtail an Ebola virus disease (EVD) outbreak was found to effectively interrupt transmission from the first generation of isolated contacts. Further, the survival rate of patients hospitalised with EVD was significantly increased by this strategy, due to improved timeliness of case detection.

**HOW THIS STUDY MIGHT AFFECT RESEARCH, PRACTICE OR POLICY**

- These findings demonstrate the importance of a community-based approach in implementing contact isolation and have relevance for policy choices regarding whether to isolate contacts or quarantine entire populations, which is particularly pertinent in resource-limited settings. Applied in various infectious disease contexts, a community-based contact isolation strategy can save lives of isolated contacts that develop infection and also prevent onward transmission to others.

## Introduction

The 10th Ebola virus disease (EVD) outbreak in the Democratic Republic of Congo (DRC) was declared on 1 August 2018 in North Kivu province’s Mabalako Health Zone (HZ).^1^ This outbreak—the first recorded in the Eastern part of the country—quickly reached large urban cities, before spreading to two other provinces, Ituri and South Kivu.

The Province of North Kivu is home to 6.6 million people of which 3.2 million live in extreme poverty. Around 2.5 million people live in the largest urban areas of Goma (around 1.2 million), Butembo (690 000) and Beni (570 000).^2^ Insecurity in the region (with over 120 armed groups active) triggered mass population movements with more than a million internally displaced people and made containment of this 10th outbreak much more challenging.^3 4^ As result, this outbreak became the second largest EVD outbreak globally, after the 2013–2016 West Africa Ebola Epidemic^5^ with a total of 3481 cases (3323 confirmed and 158 probable) and 2299 deaths were recorded.^1^

Control of EVD outbreaks has been found to be achievable through a mixed approach involving risk communication and community engagement (RCCE), early case detection, rapid isolation and care, contact tracing (CT), and the safe and dignified burial of deceased confirmed or suspected cases.^6 7^ The biological features of the Ebola virus, requiring contact with body fluids for a possibility of human-to-human transmission, place the notion of contact at the centre of the interruption of such virus-driven outbreaks.^8^ Therefore, CT is among the key EVD control measures, consisting of the identification and listing, tracing (ie, locating and establishing initial contact), and finally, regular follow-up.^9^ The core aim is to limit the spread of the infectious disease by offering early support and care as well as isolation if the contact develops disease.^10^

Accordingly, during the 2013–2016 West Africa EVD Epidemic—in which more than 28 000 cases were documented—CT was implemented as a key component of the surveillance pillar to prevent further transmission.^11 12^

However, poor performance was identified as one of the principal weaknesses of the response.^13^ For example, CT was successfully performed for only 26.7% of all EVD cases in Liberia, leading to the detection of just 3.6% of new cases^12^ (this is despite the fact that CT was less logistically complex because quarantine was enforced through the presence of army personnel and police officers, which theoretically ensured contacts remained in place at all times).^6^ This enforced quarantine was likely counterproductive and may have led to negative public health behaviours, such as hiding bodies or sick persons, and not seeking healthcare. This suggests that epidemic control interventions rooted in RCCE, social acceptance and local practices may be a more effective alternative.^14–16^ In addition, CT becomes extremely challenging and impractical beyond the early stages of large and rapidly expanding outbreaks, during which time the number of contacts grows exponentially and overwhelms the capacity to respond.^17^ Recent analyses indicate that the prevention of new clusters of cases may have been more effective at bringing that epidemic to an end than the reduction of secondary infections achieved through enhanced surveillance activities such as CT.^18^

In the 2018–2020, EVD outbreak in Eastern DRC, where more than 250 000 contacts were recorded,^1^ public health performance indicators were initially poor. This included many community deaths, poor CT^9^ and delays between symptom onset and case isolation.^19^ In response to the persistence of the outbreak, a number of initiatives and strategies were pursued, including the decentralisation of interventions in the health areas (health area approach), the establishment of a unit to search for lost to follow up contacts, and the contact isolation of contacts (Nota Bene: we use the term ‘community-based contact isolation ’ as distinct from ‘quarantine’, box 1).

Box 1 **Defining ‘community-based contact isolation’ as distinct from quarantine**

This manuscript examines a community-based isolation of EVD contacts, which in this manuscript is different from enforced quarantine. Quarantine is usually understood to be an enforced mechanism, whereby a legal obligation is impressed on a contact that may go on to develop infection. This can also include police or military enforcement, either through spot checks or on-site guarding.^49^

The concept of community-based contact isolation (as used in this manuscript), on the other hand, is a voluntary mechanism, where contacts are advised, encouraged, supported, and even incentivised—but not obliged—to self-isolate. It builds largely on Rosenthock’s health belief model including the perceived susceptibility; perceived severity and perceived benefits of the intervention.^50^

The specific design and strategy used in the 2018–2020 EVD outbreak in DRC is discussed further later in this manuscript.

DRC, Democratic Republic of Congo; EVD, Ebola virus disease.

The lack of previous studies on community-based contact isolation precluded the use of this strategy in the early stages of this outbreak as the effectiveness of such strategies has been demonstrated only in modelling studies on the impact of quarantine.^16 20–22^ Furthermore, evidence concerning the acceptability of such an intervention was deemed necessary, as previous efforts to control EVD spread have often resulted in clashes and conflict due to the fact that outbreaks often overlap with other major community needs that are neglected by political authorities. This is a particular risk when control measures do not align with local practices and expectations. In one instance in Womey village, Nzerekore prefecture, local inhabitants interpreted Ebola responders disinfecting households with bleach sprays as spreading EVD, leading to the killing of members of the Ebola response team.^23^ Further issues included inadequate public health messaging, distrust of those providing the health messages, political instability and regional conflict.^24 25^ Taken together, this allowed EVD to spread and kill thousands, when early containment could possibly have been within reach.^26^

This study aimed to provide evidence of the effectiveness of a contact isolation strategy that is more focused on contact participation, and community engagement that could be implemented in future epidemics to rapidly mitigate onward transmission.

## Methods

### Study design

A quasi-experimental (ie, non-randomly assignment) study was designed to compare isolated contacts to those who were not isolated (see ‘description of the intervention’ for definitions). As this intervention was implemented in support of the response to the EVD outbreak, participant recruitment was organised through existing Ebola response mechanisms at the initial stage of alert response: whenever a new case was confirmed and reported, a psychosocial team (a team including those trained in psychological and social first aid) visited the person and his/her family to deliver the result. The surveillance team then completed a case investigation and updated the contact list.

At this point, in collaboration with the RCCE team, the study was explained to contacts to obtain their informed consent. When fully oriented and consent was provided, contacts were assigned to one or other group by the intervention team (ie, field epidemiologists), considering the rapid risk assessment based on the type of contacts.^27^ Since we could not confine all contacts given their number and geographical dispersion, priority was given to high-risk contacts, an approach taken in Spain with contacts of the first secondary case of the 2014–2016 Ebola epidemic that occurred outside Africa.^28^ However, if a high-risk contact did not consent to isolate, they were placed in the non-isolated group.

A contact was defined as a person who is currently asymptomatic but had physical contact with an EVD patient within the past 21 days. Physical contact could be proven or highly suspected, such as having shared the same room or bed, cared for a patient, touched body fluids or closely participated in a burial (eg, physical contact with the corpse). A high-risk exposure was defined as a percutaneous or mucous membrane exposure to, or direct skin contact with blood or other body fluids of an EVD patient or corpse without appropriate personal protective equipment. A low-risk exposure was defined as a household contact that was not involved in providing care to, or having close contact with, an EVD patient in healthcare facilities or in the community that was not otherwise characterised as a high-risk exposure.^29^

### Description of the intervention

Intervention group participants were isolated in specific community sites of their preference (ie, either households or rehabilitated structures, defined as a transitional facility where living conditions have been improved to allow several individuals to stay and live there temporarily, including the installation of additional tents) for the 21-day follow-up period. In community site settings, certain contacts were grouped together on the same site, usually within their own households, thereby separating them from the rest of the community. Additional tents for contacts (to reduce contact with each other) as well as toilets, water and a solar electricity supply system were added in some areas. The use of individual utensils was encouraged (spoons and bowls were distributed). Hygiene measures were strengthened by distributing soap, hydroalcoholic solutions and installing multiple hand hygiene units to minimise cross-contamination risk. Psychosocial support was delivered according to the interagency standing committee guidelines on mental health and psychosocial support in emergency settings^30^ and financial support provided to approximately compensate for loss of income. Risk communication, awareness and sensitisation were delivered on a daily basis to isolated contacts. As part of community engagement efforts, the security services were explicitly excluded from the process, despite the outbreak occurring within a conflict zone.

**Figure 1:**
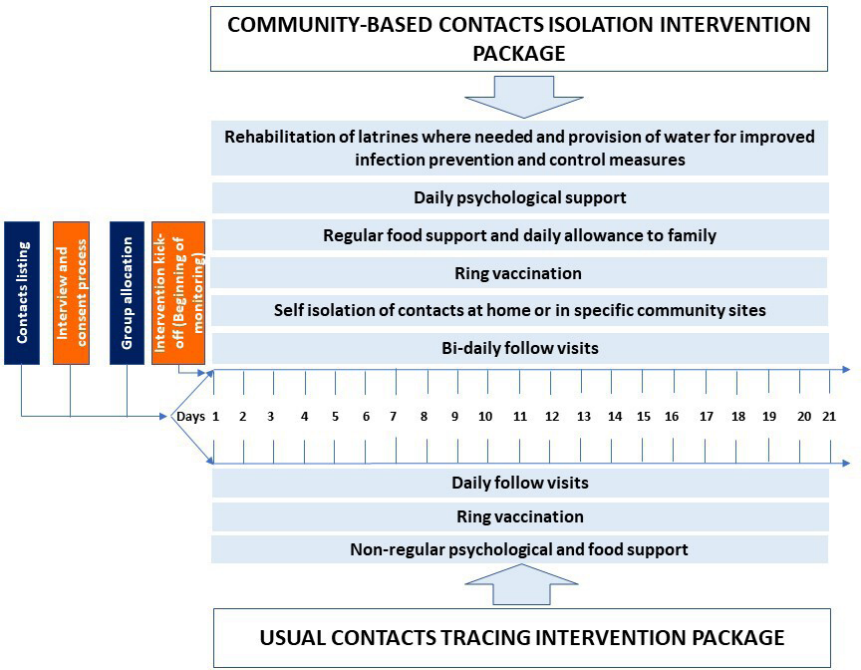
Community-based contact isolation study protocol, Beni and Mabalako sub-coordination, Democratic Republic of the Congo, June 2019–May 2020.

Meanwhile, comparison group participants underwent CT without isolation and were allowed to continue their daily activities while receiving non-regular psychosocial and food support. In both groups, daily follow-up of contacts was undertaken for a period of 21 days from the date of last contact with the index case (defined for this context as the case that led to the contacts under investigation) and vaccination performed according to the same strategy (‘ring vaccination’) (figure 1).

The community contact isolation strategy was designed in line with various principles that guided preparation for, and implementation of, CT^31^:

- Acceptance through community engagement: All affected families were actively engaged and the rationale for contact isolation and measures being taken explained (ie, isolation and prompt treatment of suspected patients, vaccination of contacts, protection of other family members and compensatory measures in terms of lost economic gain at the family level). Influential family members, local government or religious leaders were engaged to support this engagement.
- Listen to and act on the needs and concerns expressed by communities: The strategy was guided by community feedback, adapting the implementation of activities accordingly (eg, daily meal menus were suggested by contacts themselves and supported by the intervention team). The choice of contact isolation site was not imposed. Communities were welcome to express any concerns about contact isolation, and the strategy could be adapted accordingly.
- Flexibility: The strategy was adapted: to local conditions (eg, urban vs rural villages); the relative availability of contact isolation sites (especially in urban areas); and consideration of the choice of people to confine.
- Improved living conditions: Transmission of EVD often occurs in areas with poor access to water, sanitation and hygiene. Therefore, the strategy sought to improve these conditions by providing additional latrines and water supply to contact isolation sites in the respect of infection, prevention and control protocols.
- Implementation by local staff: All work to set up and/or adapt the contact isolation sites (eg, construction of toilets, installation of water tanks, installation of electrical panels, construction of fences, guarding of sites) was entirely performed by local staff, who were financially compensated for their work.

### Setting

**Figure 2:**
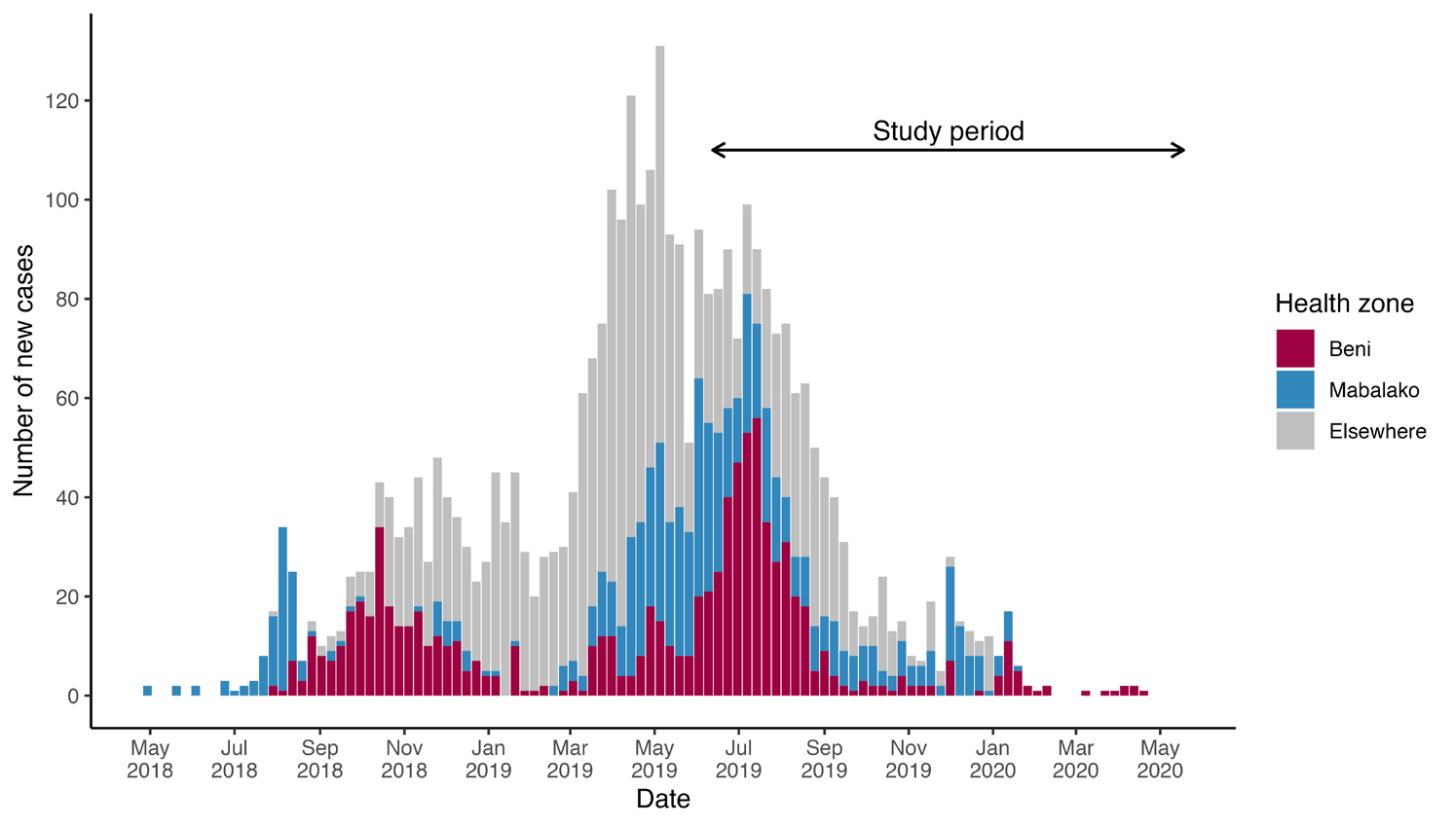
Evolution of the weekly number of confirmed and probable cases of Ebola virus disease by Health Zone, Democratic Republic of the Congo, May 2018–June 2020

The intervention was implemented in two HZs (Beni and Mabalako), from 12 June 2019 to 18 May 2020. While the outbreak was short-lived in some locations, these two HZs experienced continuous transmission over the epidemic’s 2-year duration and were the first and last two HZs to report confirmed cases, respectively (figure 2).

### Data sources and measurement

Data were collected from the investigation forms of EVD alerts, suspected and confirmed cases, individual contact listing and monitoring sheets including vaccination status, and inpatient records by field epidemiologists and data managers. Intervention group participants were compared with the comparison group over the same period.

As a measure of effectiveness, the primary outcome was measured as the reproduction number (*R*, the average number of secondary cases generated from index cases) in the two groups.^32^ The first known recorded contacts that were included in the study were considered the first generation. Confirmed cases among this group were considered the primary confirmed cases. The second generation corresponds to contacts of the primary confirmed cases. Confirmed cases from this generation were considered the secondary confirmed cases.

Secondary outcomes included the successful follow-up rate of contacts in the two groups. the delay from symptom onset to isolation and case management and the case fatality rate (CFR).

### Statistical methods

Being integrated into the outbreak response strategy, there were no sample size targets or limits, with the study continuing until the last contact completed the 21-day followed up.

Both primary and secondary outcome measures were summarised using simple descriptive statistics including mean, SD and percentage. Outcome measures were tested for differences between isolated and non-isolated. Comparisons between means were tested using the two-sample t-test, and differences between frequencies were tested using the χ^2^ test. The Mantel-Haenszel test was used to test the overall difference between the intervention and comparison group. Logistic regression analyses were performed to assess predictors of death among confirmed patients. Statistical significance was defined as p<0.05 (two sided). R V.4.0.2^33^ and STATA V.14.1 (Stata) were used to perform different analyses.

### Patient and public involvement

Opinions of the health district management team, local leaders, local political and administrative authorities, and community members were obtained and integrated to improve the intervention package before the initiation of the study.

## Results

### Participants and descriptive data

**Figure 3:**
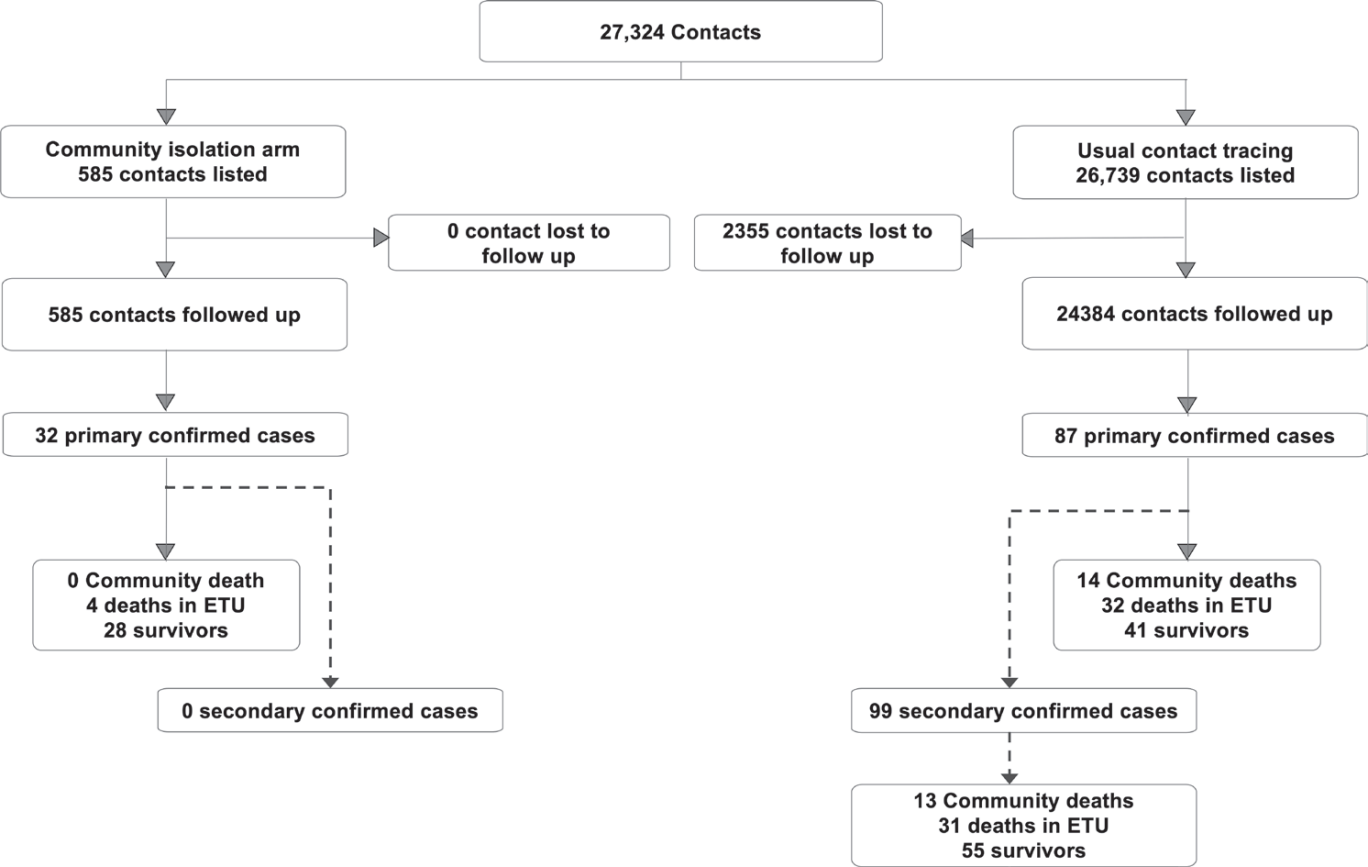
Flow diagram of the progress of contacts and their outcome through the intervention and comparison arms of the study, Beni and Mabalako sub-coordination, Democratic Republic of the Congo, June 2019–May 2020. ETU: Ebola Treatment Unit

A total of 27 324 contacts met the eligibility criteria and were included in the study (figure 3); 585 contacts underwent contact isolation while 26 739 did not. The characteristics of the two groups were quite similar regarding gender (p=0.346), but intervention group were slightly older with regard to age (median of 25.9 years vs 24.3 years; p=0.013). However, the risk of exposure (determined by the nature of the relationship with the index case and the type of contact) was significantly higher in the intervention group, as high-risk contacts were prioritised for isolation (table 1).

**Table 1:** Baseline characteristics of study participants according to intervention arm, Beni and Mabalako sub-coordination, Democratic Republic of the Congo, June 2019–May 2020.

Characteristic	Intervention arm (community contact isolation)	Comparison arm (standard community contact tracing)	P value
Age (years)	n=520 250.9 (24.6; 27.2)	n=24586 240.3 (24.1; 24.5)	0.013
Gender			
Male	301	13140	0.346
Female	283	13 414	
Type of contacts			
1	58	4871	
2	113	7130	<0.00001
3	225	6331	
4	105	2794	
Relation with the index case			
Nosocomial	69	882	
Household family members	168	3817	<0.00001
Community	214	13 400	

### Primary outcome

A total of 32 primary confirmed cases resulted from the 585 isolated contacts (54.7‰) compared with 87 of the 24 384 non-isolated contacts (3.57‰). There were no secondary confirmed cases arising from the 32 primary confirmed cases in the intervention group, whereas 99 secondary confirmed cases arose from the 87 primary confirmed cases from the comparison group.

### Secondary outcomes

**Figure 4:**
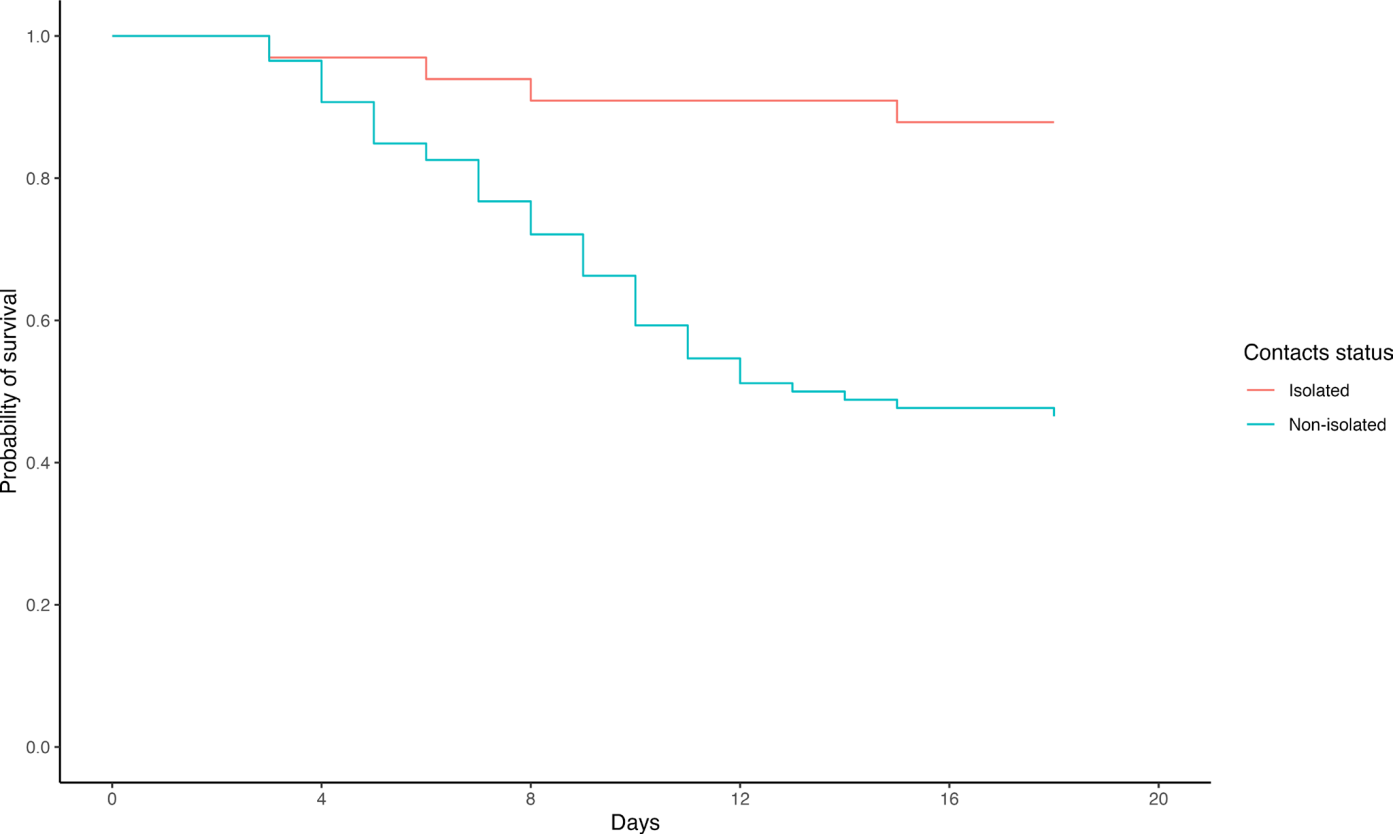
Kaplan-Meier survival curves for confirmed Ebola virus disease case-patients among the intervention (isolated) and comparison (non-isolated) groups, Beni and Mabalako sub-coordination, Democratic Republic of the Congo, June 2019–May 2020.

There were significant differences between the intervention and comparison groups in all the secondary outcomes explored (table 2). The average delay between symptom onset and case isolation was shorter (1.3 vs 4.8 days; p<0.000), CFR lower (12.5% vs 48.4%; p<0.000) and vaccination uptake higher (86.0% vs 56.8%; p<0.000) in the intervention group compared with the comparison group. A significant difference was also found between intervention and comparison groups in survival rate at discharge of hospitalised confirmed patients (87.9% vs 47.7%, respectively; p=0.0004) (figure 4).

**Table 2:** Assessment of intervention and comparison groups according to primary and secondary objectives, Beni and Mabalako sub-coordination, Democratic Republic of the Congo, June 2019–May 2020.

Characteristic	Intervention arm (community contact isolation)	Comparison arm (standard community contact tracing)	P value
No of secondary cases	32	87	–
Average delay between symptom onset and isolation (days)	1.3 (n=32)	4.8 (n=86)	0.0000
Case fatality rate	12.5% (n=32)	48.4% (n=186)	0.0001
Vaccination uptake among contacts	86.0% (n=585)	56.8% (n=26 739)	0.0000

The multivariable regression analysis showed that neither age, gender nor vaccination status had an impact on CFR in confirmed cases admitted to Ebola Treatment Centres. However, the risk of death was more than six times greater in the comparison group compared with the intervention group (table 3).

**Table 3:** Results of multivariable analysis of predictors of death among confirmed cases, Beni and Mabalako sub-coordination, Democratic Republic of the Congo, June 2019–May 2020.

Multivariable analysis			
	OR adjusted	95% CI	P value
Contact isolation			
Isolated (ref)	1		
Non-isolated	6.45	(1.46 to 28.38)	0.01
Sex			
Male (ref)	1		
Female	1.02	(0.44 to 2.38)	0.95
Vaccination			
Vaccinated (réf)	1		
Non vaccinated	1.04	(0.31 to 3.47)	0.94
Age group			
0–35 years (ref)	1		
36–65 years	0.31	(0.01 to 5.64)	0.43
66 years plus	0.71	(0.05 to 8.88)	0.79

## Discussion

To the best of our knowledge, this is the first comparative study conducted during an ongoing EVD epidemic to demonstrate the effectiveness of contacts isolation. The approach was based on enhanced risk communication and individual commitment without any obligation or constraint on EVD contacts, unlike enforced quarantine, and therefore, is a less restrictive contact isolation strategy.

The 2018–2020 eastern DRC outbreak lasted nearly 2 years despite the availability of effective vaccine^34^ and therapeutics^35^ from the outset of the response. Public health performance indicators were poor, with increasing community deaths, poor CT (indicated by the high number of cases that had no known contacts), and delays between symptom onset and isolation.^9 19 34^ The change in strategy to adapt to a difficult context was necessary, and led to a rapid and drastic reduction in transmissibility which reduced incidence and helped bring the outbreak under control.^3^ The contact isolation strategy was then implemented to avoid a new spread of the epidemic, especially as the security situation was more critical.

Although implemented after the peak of the epidemic, this strategy played an important role in accelerating control as it contributed to rapidly stopping the remaining transmission chains. The overall comparison between intervention and comparison group showed a significant difference in the outcome indicators, namely the reproduction number, CFR, delay from symptom onset to case isolation and vaccination uptake among contacts. Moreover, for all confirmed cases from the intervention group, the average delay between the date of vaccination and the onset of symptoms was less than ten days, meaning that all these cases were already in incubation at the time they were vaccinated. This implies that, even vaccinated, these contacts could have contaminated other people if they were not isolated as vaccine is effective when administered early to contacts. Ring vaccination is known to be most effective in contacts of contacts (second ring) than contacts (first ring), as some contacts are often exposed several days before the confirmation of their primary case.^36^ The rapid control of the recent Sudan Virus Disease outbreak in Uganda,^37^ in which no vaccine was available for contacts, may be associated with the isolation of high-risk contacts applied by the health authorities especially when confirmed cases were reported in Kampala.

Survival analysis showed a higher survival of confirmed cases from the intervention group than the comparison group. The higher survival rate may be attributable to the early detection of confirmed cases in the intervention group, as supported by the shorter delay from symptom onset to case isolation in this group. This delay was reported as one of the factors associated with EVD death in Guinea during the 2013–2016 West Africa Ebola Epidemic.^38^ This survival difference is likely not treatment-related as there was no difference between the two groups. All hospitalised patients received one of the two specific molecules that had already been validated in the first stage of a clinical trial conducted during the same epidemic.^35^ Finally, the security context is unlikely to have had an impact as both groups were in the same localities, and therefore, subject to the same conditions.

In contrast to the traditional quarantine, the community-based contact isolation strategy applied in eastern DRC from June 2019 to May 2020 is unique in its method of implementation, and the acceptance by those concerned. First, it only involved contacts, as opposed to the general population (ie, it was targeted). Second, it was designed by a multidisciplinary team including social scientists. The methodological approach based on community participation and engagement, inclusion of participants’ expectations and the support of psychosocial experts at all levels mitigated the negative impact of contact isolation on mental health. No cases of mental disorders were reported among the isolated population in contrast to what is reported in the quarantines during COVID-19.^39^ The implementation was also guided by WHO recommendations on quarantine, which state that if a decision to implement it is taken, the authorities should ensure that those in quarantine are adequately supported. This means adequate food, water, protection, hygiene and communication provisions; infection prevention and control (IPC) measures; and the monitoring of quarantined persons.^40^ Introducing quarantine measures early in an outbreak may delay the introduction of the disease to a new country or area and may delay the peak where local transmission is ongoing. However, if not implemented properly, quarantine may also create additional sources of contamination and dissemination of the disease.^41^ In addition, quantitative models have also shown that quarantine and symptom monitoring of contacts with suspected exposure to an infectious disease are key interventions for the control of emerging epidemics.^42^

The novel community-based contact isolation strategy drawing on these concepts of quarantine was applied during the 2018–2020 North-Kivu outbreak and has great potential for future outbreaks for which CT and isolation is recommended, including, but not limited to, EVD. This includes Marburg Virus Disease, an emerging and increasingly frequent viral haemorrhagic fever in Africa, caused by a virus of the same family of Filoviridae as Ebola Virus.^43 44^ For the first time since 1975, two concurrent outbreaks of Marburg Virus Disease occurred in Africa in 2023, in Equatorial Guinea and Tanzania.^45^ In Equatorial Guinea, more than 200 people were quarantined, and movement was restricted along its border.^46^ While the availability of the EVD vaccine has markedly reduced transmission during EVD outbreaks, there is a possibility of relapse up to 5 years after infection.^47^ This reinforces the need to consider, strengthen and more broadly apply community contact isolation strategies for the rapid containment of future outbreaks. This will require trust from affected populations, which should not be taken for granted. However, the strategy itself can also serve to engender this trust, and therefore, also strengthen the positive effect of other interventions requiring this trust, which includes all five core pillars of EVD response (ie, case management, case finding and CT, IPC, safe and dignified burial, and risk communication and community engagement).^7^

### Limitations

As allocation to the group was not random, but rather based on the risk associated with the type of contact, some high-risk contacts who did not want to be isolated may be motivated to falsely report not having been in close contact with a confirmed or probable case. This potential bias is likely to be mitigated by the validation of the group assignment by field epidemiologists. Moreover, the impact is not significant as it would to some extent rebalance the level of risk that was estimated to be higher in the intervention group.

The size of the intervention and comparison groups was vastly different, with the comparison group being approximately 45 times the size of the intervention group. This reflects the radical, more costly and somewhat experimental nature of the contact isolation strategy, which resulted in only a small proportion, including the most high-risk, contacts being proposed for the intervention. However, our analysis of baseline characteristics revealed the groups to be broadly similar in most sociodemographic characteristics. Furthermore, the imbalance in participant numbers between groups would not have negatively impacted the statistical power of the assessment, which was sufficient in the small of the two groups.^48^

## Conclusion

The rapidly evolving nature of the 2018–2020 Kivu epidemic has shown that unaddressed EVD transmission chain can escalate into further (and lethal) transmission. Therefore, CT strategies—including in areas with such weak health systems and conflict—should consider methods of rapid identification and isolation of contacts accompanied by a range of supportive interventions and with community engagement. This study has shown that doing so can help interrupt disease transmission when done using the community-based contact isolation approach. More than just saving lives through limiting onward transmission, it also has the added advantage of engaging affected individuals, as well as key and trusted community actors, which can help to engender and maintain trust in the response. Further, it limits the need to use more costly forms of containment such as enforced quarantine or regional lockdowns. In short—for the ease of the strategy’s implementation, the integration of social sciences, the engagement of affected communities and trust built among them (which is itself key to the overall effectiveness of an outbreak response)—the community contact isolation strategy should be considered on a case by case basis as a potentially effective and efficient method of saving lives.

## Data Availability

Data are available on reasonable request.
